# A Randomized Controlled Trial of Intralesional Glucocorticoid for Treating Pretibial Myxedema

**DOI:** 10.14740/jocmr2303w

**Published:** 2015-09-25

**Authors:** Changgui Lan, Can Li, Wei Chen, Xiaofeng Mei, Jing Zhao, Jie Hu

**Affiliations:** a Department of Dermatology, China National Nuclear Corporation 416 Hospital, No.4, Er Huan Lu Bei Si Duan, Chengdu City, Sichuan Province, China

**Keywords:** Pretibial myxedema, Randomized controlled trial, Glucocorticoid, Efficacy, Safety, Recurrence

## Abstract

**Background:**

Pretibial myxedema (PTM) is an uncommon dermopathy associated with autoimmune thyroid diseases. Now it is thought to be autoimmune and its treatment with glucocorticoid is helpful. However, it has not been evaluated.

**Methods:**

A prospective randomized controlled trial was performed in 110 patients with PTM to evaluate the efficacy and safety of triamcinolone acetonide with intralesional injection once every 3 days and once every 7 days. Randomization was performed with drawing lots and it was also stratified according to variants of PTM lesions. In the follow-up, recurrent rates were observed. The SPSS Statistics 17.0 Software was used in the statistical analysis.

**Results:**

The complete response rates were 78.2%, 83.6%, and 87.3% in regimen 1 and 50.9%, 89.1%, and 90.9% in regimen 2 at 3 weeks, 7 weeks and the end of therapy, respectively. Regimen 1 had an earlier efficacy than regimen 2, but at 7 weeks and end of therapy, there were no differences between two regimens. The majority of non-severe variants got complete response but severe variants did not. The adverse reaction rates in regimen 1 were higher and earlier than those in regimen 2. Adverse reaction occurring time in regimen 1 was shorter than that in regimen 2. Recurrent rates were 31.25% and 32% in regimens 1 and 2 at 3.5-year follow-up.

**Conclusions:**

For its autoimmune, hyperplasia and disabled features, early treatment of PTM with glucocorticoid is necessary to get complete response. Dosage and frequency of intralesional steroid injection and lesional variants influence the efficacy of PTM. Once every 7 days is a better regimen.

## Introduction

Localized myxedema or thyroid dermopathy is an uncommon dermopathy associated with autoimmune thyroid diseases [1], first reported by Hektoen in 1895 [2]. Its prevalence is 1.6% in thyroid diseases in China [3]. Commonly located at the pretibial area, it is also often known as pretibial myxedema (PTM). In fact, PTM is characterized by non-pitting thickening of local skin with autoimmunity, hyperplasia and infiltration [4-6]. Its treatment has been a problem in dermatology and endocrinology. Searching the “pretibial myxedema and treatment” in Medline database, there have existed lots of reports about the treatment of PTM such as intralesional injection of thyroid hormone, hyaluronidase, plasmapheresis, surgery, glucocorticoids, and so on. The majority of them are case or case series reports and most modalities are of glucocorticoids. Since the glucocorticoid was first used to treat PTM in 1953 [7], the modalities of glucocorticoids are involved in systemic, intralesional and topical usages with different dosages and frequencies. However, according to our experience, intralesional usage of glucocorticoid is seemly more helpful to PTM but it has not been evaluated. In order to evaluate the efficacy and safety of intralesional injection of glucocorticoid for treatment of PTM, we designed and performed the clinical randomized controlled trial with two different frequency schedules.

## Patients and Methods

### Study patients

The patients were eligible if they met the diagnostic criteria and were not excluded from exclusion criteria. The diagnostic criteria [8] of PTM had: autoimmune thyroid diseases or the history of autoimmune thyroid diseases; localized non-pitting swelling, or nodule, or plaque, or mixture, or elephantiasis; histopathology showing most marked features of mucinous degeneration and positive Alcian blue staining; apart from the lesions caused by infection, radiation therapy, venous stasis and other mucinoses. Before patients with PTM entered into the trial, they should be excluded if the patients had one of the following: younger than 16 years old; associated with high blood pressure or hyperglycemia, liver or kidney function insufficiency; immunodeficiency, or mental disorders; shorter than 3 months after glucocorticoid was used; pregnant or lactating women; no written informed consent form.

### Study design

In the randomized controlled trial, eligible patients were randomly assigned in a 1:1 ratio to receive intralesional injection of triamcinolone acetonide acetate once every 3 days in regimen 1 and once every 7 days in regimen 2. The multipoint intralesional injection was performed to distribute the dosage of glucocorticoid in the whole lesion at each session. Seven injection sessions were as a therapy course. After ending therapy, follow-up was performed at 1, 6, 12, 24 and 42 months to observe recurrent rates of both regimens.

The trial as a project was set up by Health Department of Sichuan Province in China (No. 080141) and it was approved by research ethics committees in Chinese National Nuclear Corporation (CNNC) 416 Hospital.

### Randomization and stratification

**Figure 1:**
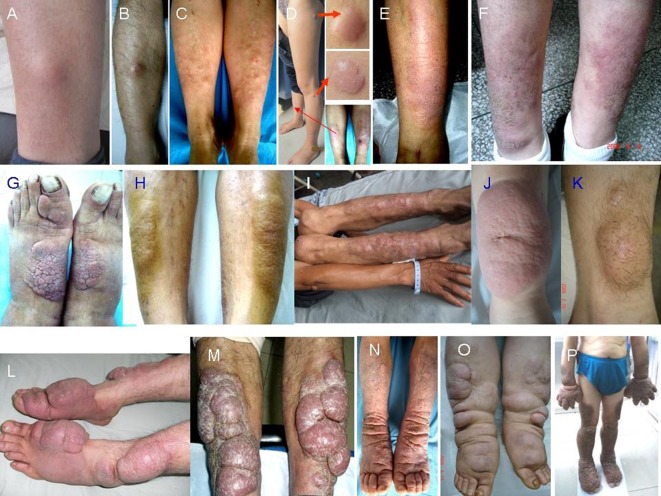
Morphology of PTM variants. Nodules: single erythema and nodule at the unilateral pretibial region (A, B), multiple erythema and nodules at bilateral pretibial regions (C), red nodules at the scar sites (D); diffuse swelling: erythema and non-pitting swelling at the unilateral extensor of a lower leg (E), erythema and non-pitting swelling at bilateral extensors of lower legs (F); plaques: plaques with papillary and polypoid appearance at bilateral dorsa of feet and a toe (G), plaques with peau d’orange appearance at bilateral extensors of lower legs (H); mixtures: single nodule at right elbow and multiple nodules and plaques at bilateral extensors of lower legs (I); giant plaques: a single red plaque with significant protrusion above the normal skin and infiltrate into subcutaneous tissue at the unilateral extensor of a lower leg (J), a giant plaque with hypertrichosis hair and infiltrate into adipose (K); tumors: multiple ball-like red tumors at bilateral extensors of lower legs and dorsa of feet and toes (L), lobulated ball-like tumors with scales and tension appearance at bilateral extensors of lower legs (M); elephantiasis: extensive, indurated, non-pitting swelling and infiltration with peau d’orange appearance of multiple waxy nodules and plaques and hyperpigmentation from lower legs to feet and toes on both lower legs (N), extensive, indurated, non-pitting swelling and infiltration with multiple waxy nodules and plaques from lower legs to feet and toes (O), extensive, indurated, non-pitting swelling and infiltration with papillary and polypoid appearance and hyperpigmentation from one-third of upper legs to feet and toes on both lower extremities and from half of forearms to hands and fingers on both upper extremities (P).

Randomization was performed with drawing lots and it was also stratified according to variants of PTM lesions (Fig. 1). The non-severe variants included the nodule, plaque, diffuse swelling and mixture. The severe variants included tumor, giant plaque and elephantiasis. The progressing lesion within < 1 month was defined as active, or as stable.

### Protocol of multipoint injection

Geometric areas of lesions were firstly measured at each patient before injection. The dose of triamcinolone acetonide acetate injection (50 mg/5 mL) was calculated according to 8 mg triamcinolone acetonide acetate per 2-cm-diameter circle area at each session (experienced dose), but the total dose was not more than 100 mg at each session in a patient. The dose of triamcinolone at each session was evenly distributed by multipoint intradermal injections in 1 mL syringe with 0.45 × 16 RW·LB needle [9]. Triamcinolone acetonide (0.8 mL, 8 mg) was added with 2% lidocaine hydrochloride 0.2 mL at each point. The injections started along the borderlines of lesions and then gradually spread to the centers of lesions in the subsequent treatment sessions after the indurated lesions became softer and thinner. When swelling lesions disappeared and the skin became as thin as normal, the injection was stopped. We discontinued the injection if the lesion did not improve for two continual injection sessions or severe adverse reactions occurred.

### Outcome measures and follow-up

In the trial, patients were assessed at 3 and 7 weeks after the first injection and at the end of treatment. Lesional geometric areas were obtained from direct measurement. Lesional skin depth (epidermis + dermis) was multipointly detected with color Doppler ultrasound iU22 (Philips Ultrasound Inc., USA) [10] and lesional skin depth was the average of multipoint depth. Lesional depth = lesional skin depth before therapy (measured value) - normal skin depth after therapy (measured value). Lesion volume = lesional geometry areas (cm^2^) × lesional depth (cm). Efficacy outcomes included the decreased amounts of lesions, the time and the rate to get complete response. Responses to therapy were recorded as complete response when the reduction of lesion volume was 100%, major response when the reduction ≥ 50%, < 100%, partial response when the reduction ≥ 30%, < 50% and no response when the reduction < 30% or no reduction or even increase. The safety assessment was performed by the rate and the time of adverse reaction occurrence. In the follow-up, patients with complete response were assessed by the recurrent rate and the time.

### Statistical analysis

The SPSS Statistics 17.0 Software was used in the statistical analysis of the trial. Quantitative data such as age, course, the area, depth and volume of lesions, and time were analyzed by *t*-test, paired samples *t*-test, independent samples *t*-test or one-way ANOVA. Qualitative data such as sex, occupation, response rate, and recurrent rate were analyzed by Pearson’s Chi-square and Mann-Whitney U tests. Influence of PTM variants in the clinical effects of steroid was analyzed by Cox’s proportional hazards regression model. P ≤ 0.05 was statistical difference and P ≤ 0.01 was significant difference.

## Results

Characteristics of participants

**Figure 2:**
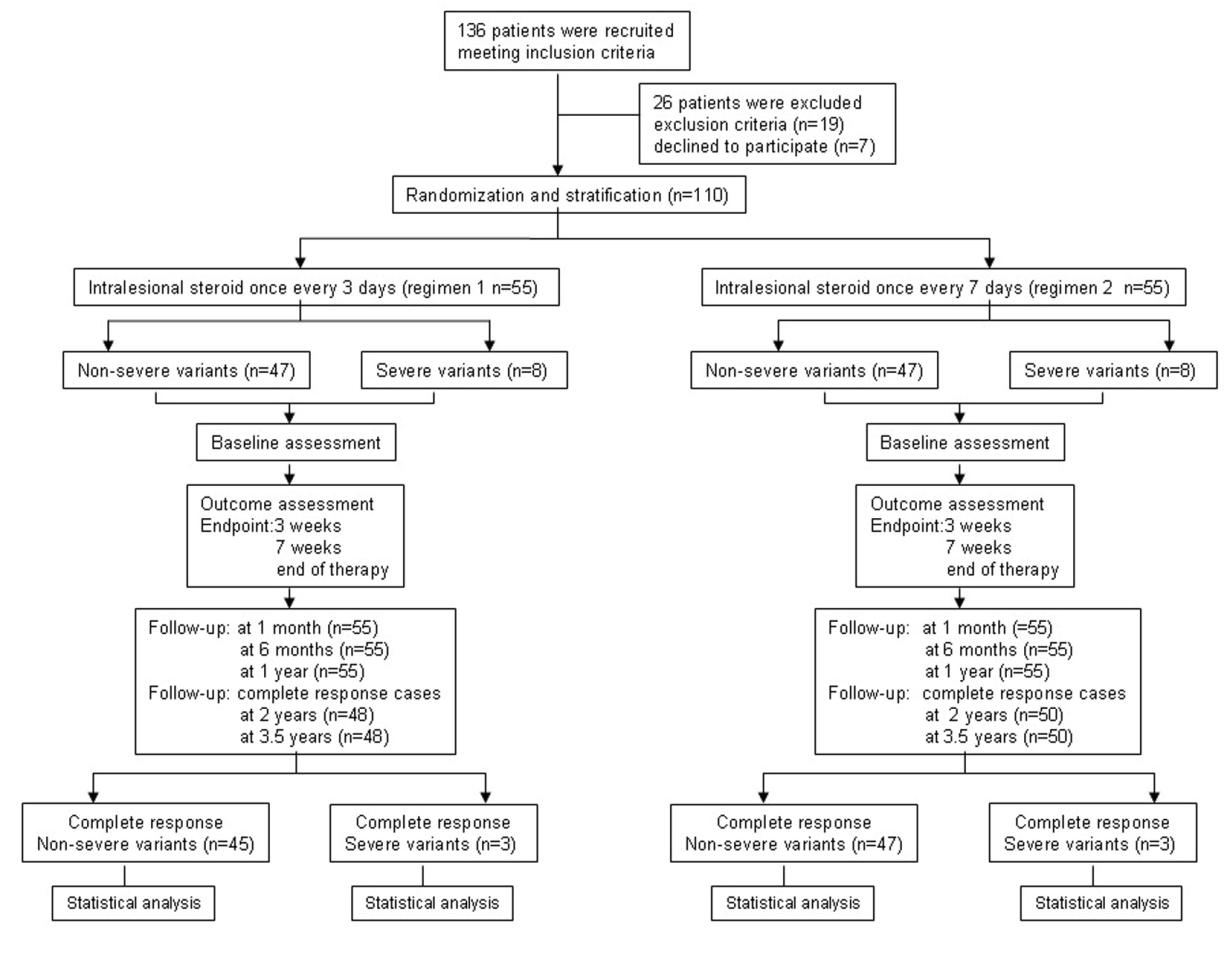
Flowchart of the trial process.

From June 23, 2009 to October 25, 2010, a total of 136 patients with PTM were recruited at the outpatients of our Dermatology Department and Nuclear Medicine Department in CNNC 416 Hospital and 110 of them were eligible. Twenty-six patients were excluded because nine patients were with high blood pressure, 10 with hyperglycemia and seven patients preferred surgery. One hundred and ten patients in both regimens completed therapy trial and 1 year follow-up but 98 patients with complete response finished 3.5 years follow-up (Fig. 2). The demographic and clinical characteristics of the110 patients in both regimens are shown in Table 1. The average age was 44.65 (18 - 77) and 40.73 (17 - 65) years old in regimen 1 and regimen 2 respectively. The sex ratio was 0.83 (M/F). PTM was seen in Chinese farmers, workers, cadres and city dwellers, but Chinese farmers were most, 76.4% in regimen 1 and 74.6% in regimen 2. The course was 22.3 (1 - 120) months in regimen 1 and 21.7 (1 - 120) months in regimen 2 respectively. Both regimens consisted of 47/55 non-severe variant (85.5%) and 8/55 severe variant (14.5%). The most common variant was the diffuse swelling, 24 cases (43.6%) in regimen 1 and 25 cases (45.5%) in regimen 2. The tumorous variant was the least, one case (1.8%). The majority of lesions were distributed at both lower extremities. Unilateral distribution was only 2/55 cases in both regimens. Except for the most common sites at lower leg extensors, the feet, digits and hands could also be involved by PTM lesions. Active lesions were 36/55 cases and stable were 19/55 cases in both regimens. There were no statistical significances between two regimens.

**Table 1:** Demographic and Clinical Characteristic of Participants at Baseline

Characteristic	Regimen 1 (N = 55)	Regimen 2 (N = 55)	Test	P value
Age (mean ± SD), years	44.65 ± 11.47	40.73 ± 12.24	t = 1.736	0.085
Range (min - max)	18 - 77	17 - 65		
Sex ratio (male/female)	0.83 (25/30)	0.83 (25/30)	χ^2^ =0.00	1.00
Occupation			χ^2^=0.345	0.951
Chinese farmer	42	41		
Worker	1	2		
Cadre	2	2		
City dweller	10	10		
Course (mean ± SD), months	22.25 ± 24.19	21.69 ± 23.88	t = 0.123	0.902
Range (min - max)	1 - 120	1 - 120		
Numbers of lesions	2.87 ± 2.64	2.42 ± 1.20	t = 1.163	0.247
Types of lesion				
Nodule	8	13		
Plaque	6	5		
Diffuse swelling	24	25		
Mixture	9	4	χ^2^ = 8.648	0.335
Giant plaque	3	3		
Tumorous	1	1		
Elephantiasis	4	4		
Distribution of lesion				
Both lower legs	42	48		
Both lower legs and feet	6	5		
Both lower legs and right hand	1	0		
Both lower legs and left toes	1	0	χ^2^ = 6.824	0.447
Both lower legs and left foot	1	0		
Dorsum of feet	2	0		
Right lower leg	1	0		
Left lower leg	1	2		
Stage of lesion				
Active/stable	36/19	36/19	χ^2^ = 0.000	1.00

### PTM is relieved by intralesional injection of glucocorticoid

**Figure 3:**
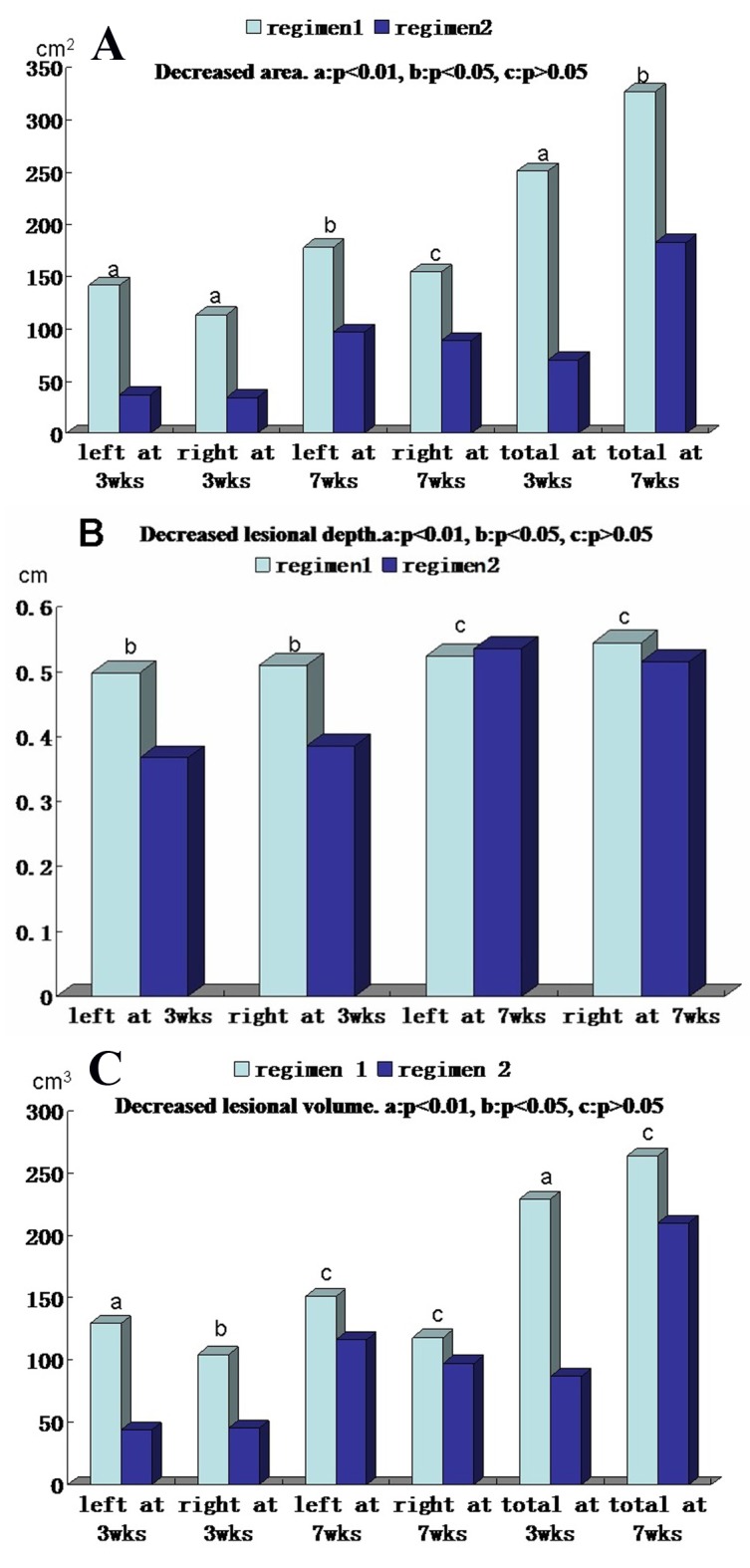
Comparison of decreased area, thickness and volume of pretibial myxedema lesions at 3 and 7 weeks after therapy between regimens 1 and 2. (A) Decreased lesional areas of left side, right side and both sides (total) at 3 weeks and 7 weeks after therapy between two regimens. (B) Decreased lesional depth of left side and right side at 3 weeks and 7 weeks after therapy between two regimens. (C) Decreased lesional volume of left side, right side and both sides (total) at 3 weeks and 7 weeks after therapy between two regimens. In the A, B and C column diagram, “a” is significant difference between two regimens (P < 0.01), “b” is statistical difference between two regimens (P < 0.05) and “c” is no difference between two regimens (P > 0.05).

**Figure 4:**
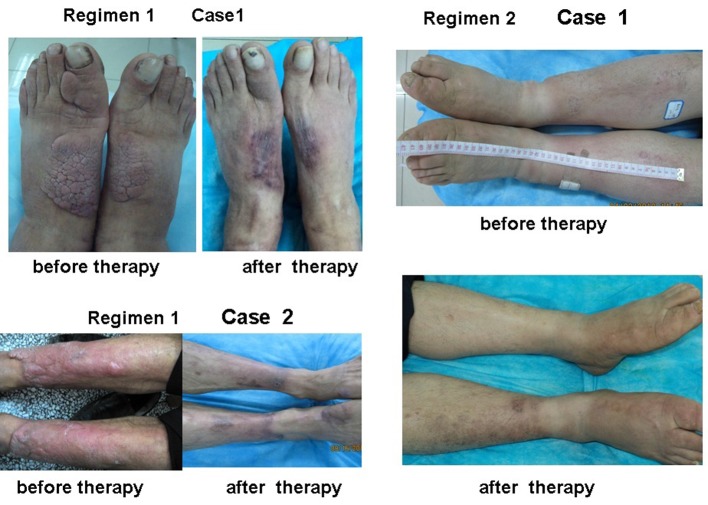
Plaque and diffuse swelling variants of PTM before and after therapy in regimens 1 and 2. Regimen 1 case 1 showed a case with PTM plaques on the dorsum of feet and first toes before and after therapy in regimen 1. Before therapy, erythematous, indurated, non-pitting, verrucous plaques were located on the dorsum of feet and first toes. After therapy, the plaques disappeared and remained erythema and pigmentation. Regimen 1 case 2 showed a case with erythematous plaques and nodules at the extensors of lower legs before and after therapy in regimen 1. Before therapy, erythematous, indurated, non-pitting plaques with nodules were located at the major area of lower leg extensors. After therapy, the plaques and nodules disappeared and remained pigmentation. Regimen 2 case 1 showed a case with diffuse swelling of lower legs and feet. Before therapy, the lower legs and feet were diffuse swelling. After therapy, diffuse swelling disappeared and remained mild pigmentation at the left lower leg.

The area, depth and volume of PTM lesions were significantly decreased at 3 and 7 weeks after first injection compared with those before therapy in both regimens (Table 2). However, the decreased amounts of the area, depth and volume of PTM lesions in regimen 1 were more than those in regimen 2 at 3 weeks after therapy began, but to 7 weeks after therapy, the decreased amounts of both side lesion areas and left lesion areas in regimen 1 were more than those in regimen 2; the rest in regimen 1 had no significant differences compared with those in regimen 2 (Fig. 3). We also observed that the thickened skin receded earlier than its erythema after glucocorticoid therapy. Sometimes the swelling or plaque completely disappeared but the erythema and pigmentation still existed (Fig. 4).

**Table 2:** Comparison of Area, Thickness and Volume of PTM Lesions Between Before and After Therapy

	Before therapy (mean ± SD)	After therapy		Paired samples *t*-test					
		At 3 weeks (mean ± SD)	At 7 weeks (mean ± SD)	Before and at 3 weeks *t* value	P value	Before and at 7 weeks *t* value	P value	At 3 weeks and at 7 weeks *t* value	P value
Left area (cm^2^)									
Regimen 1 (N = 54)	255.2 ± 381.4	113.5 ± 247.8	77.1 ± 219.4	4.818	0.000	5.308	0.000	2.704	0.009
Regimen 2 (N = 55)	172.1 ± 360.7	134.7 ± 352.1	75.2 ± 264.0	5.363	0.000	5.85	0.000	4.088	0.000
Right area (cm^2^)									
Regimen 1 (N = 54)	221.6 ± 336.2	108.2 ± 220.2	67.4 ± 189.8	4.374	0.000	4.915	0.000	3.116	0.003
Regimen 2 (N = 53)	151.7 ± 297.6	117.0 ± 282.0	62.9 ± 210.8	4.003	0.000	5.79	0.000	4.165	0.000
Both side area (cm^2^)									
Regimen 1 (N = 55)	468.7 ± 707.7	217.7 ± 461.2	141.9 ± 403.4	4.718	0.000	5.159	0.000	2.938	0.005
Regimen 2 (N = 55)	318.3 ± 646.9	247.5 ± 624.3	135.8 ± 461.8	5.014	0.000	5.968	0.000	4.173	0.000
Left skin depth (cm)									
Regimen 1 (N = 54)	0.739 ± 0.468	0.241 ± 0.337	0.216 ± 0.303	12.211	0.000	12.695	0.000	2.147	0.036
Regimen 2 (N = 55)	0.734 ± 0.340	0.367 ± 0.396	0.199 ± 0.244	11.618	0.000	15.052	0.000	4.914	0.000
Right skin depth (cm)									
Regimen 1 (N = 54)	0.764 ± 0.445	0.254 ± 0.354	0.218 ± 0.297	12.939	0.000	13.736	0.000	3.02	0.004
Regimen 2 (N = 53)	0.716 ± 0.306	0.333 ± 0.354	0.203 ± 0.241	11.426	0.000	15.676	0.000	4.354	0.000
Left lesion depth (cm)									
Regimen 1 (N = 54)	0.619 ± 0.469	0.120 ± 0.337	0.095 ± 0.303	12.223	0.000	12.695	0.000	2.133	0.038
Regimen 2 (N = 55)	0.617 ± 0.339	0.250 ± 0.396	0.082 ± 0.243	11.635	0.000	15.067	0.000	4.902	0.000
Right lesion depth (cm)									
Regimen 1 (N = 54)	0.641 ± 0.447	0.13 ± 0.354	0.096 ± 0.297	12.852	0.000	13.659	0.000	3.02	0.004
Regimen 2 (N = 53)	0.598 ± 0.307	0.2123 ± 0.356	0.083 ± 0.241	11.408	0.000	15.662	0.000	4.334	0.000
Left volume (cm^3^)									
Regimen 1 (N = 54)	191.1 ± 433.3	61.9 ± 254.2	40.1 ± 203.1	4.517	0.000	3.946	0.000	1.478	0.145
Regimen 2 (N = 55)	176.4 ± 484.7	132.8 ± 455.5	60.1 ± 237.7	4.197	0.000	3.398	0.001	2.409	0.019
Right volume (cm^3^)									
Regimen 1 (N = 54)	165.4 ± 375.7	61.2 ± 259.1	47.3 ± 210.6	5.196	0.000	4.64	0.000	-1.65	0.105
Regimen 2 (N = 53)	142.5 ± 355.0	97.5 ± 300.8	45.3 ± 174.3	3.533	0.001	3.708	0.001	2.727	0.009
Both side volume (cm^3^)									
Regimen 1 (N = 55)	350.0 ± 798.9	120.9 ± 508.3	85.9 ± 403.7	4.848	0.000	4.27	0.000	1.682	0.098
Regimen 2 (N = 55)	313.7 ± 824.5	226.8 ± 744.6	103.8 ± 402.9	3.914	0.000	3.571	0.001	2.554	0.013

### Efficacy and safety outcomes after intralesional injection of glucocorticoid

The efficacy of intralesional glucocorticoid injection treating PTM was evaluated at 3 weeks, 7 weeks and the end of therapy. The complete response rate (78.2%) in regimen 1 was higher than 50.9% in regimen 2 at 3 weeks after therapy began (P < 0.05). Two cases in regimen 1 and seven cases in regimen 2 had no response. At 7 weeks, all cases had response. In regimen 1, 83.6% cases and in regimen 2, 89.1% cases had complete response but there was no statistical difference between two regimens (P > 0.05). At the end of therapy, 87.3% cases in regimen 1 and 90.9% cases in regimen 2 obtained complete response. Ten cases had major response and two cases had partial response. All of cases had responses. There was no statistical difference between two regimens (P > 0.05). The safety of two regimens was assessed to find that all patients experienced transient pain at injection sites. The other adverse reactions included ecchymosis, infection, fragile skin, hyperglycemia, high blood pressure, Cushing’s syndrome, lower limb muscle pain and lower limb muscle weakness. Among them, hyperglycemia, high blood pressure and Cushing’s syndrome were the most common. The rate (11.7%) of adverse reactions in regimen 1 was higher than that (6.4%) in regimen 2 and the adverse reaction occurring time (17.9 ± 2.9 days) in regimen 1 was shorter than that (42.6 ± 5.7 days) in regimen 2 (Table 3).

**Table 3:** Comparison of Efficacy and Safety of Intralesional Steroid Treating PTM Between Regimens 1 and 2

	Regimen 1 (N = 55)	Regimen 2 (N = 55)	Statistical analysis	
	Intralesional steroid once every 3 days	Intralesional steroid once every 7 days	Test	P value
Efficacy				
At 3 weeks after therapy began				
Complete response	43 (78.2%)	28 (50.9%)	χ^2^ = 8.422	0.038
Major response	8	14		
Partial response	2	6		
No response	2	7		
At 7 weeks after therapy began				
Complete response	46 (83.6%)	49 (89.1%)	χ^2^ = 0.913	0.634
Major response	7	4		
Partial response	2	2		
No response	0	0		
At end of therapy				
complete response	48 (87.3%)	50 (90.9%)	χ^2^ = 0.441	0.802
major response	6	4		
partial response	1	1		
no response	0	0		
Safety				
Pain at injection sites, No. (%)	55 (100%)	55 (100%)		
Ecchymosis, No.	3	1	Z = -4.332	0.000
Infection, No.	1	1		
Fragile skin, No.	3	2		
Hyperglycemia, No.	13	7		
High blood pressure, No.	9	6		
Cushing's syndrome, No.	12	6		
Lower limb muscle pain, No.	2	2		
Lower limb muscle weakness, No.	3	3		
Average adverse reaction except pain, No. (%)	46/440 (11.7%)	28/440 (6.4%)	χ^2^= 4.78	0.029
Adverse reaction occurrence time except pain (mean ± SD), days	17.9 ± 2.9	42.6 ± 5.7	t = -24.554	0.000

### Influence of dosage and frequency in clinical effects of steroid

We observed the relationship among dosage, frequency and clinical effects of steroid in the trial. The clinical effect occurring time after injection of triamcinolone acetonide acetate in regimen 1 was 2.5 ± 0.6 (1 - 4) days, shorter than 3.1 ± 0.8 (1 - 7) days in regimen 2 and the complete response occurring time (14.9 ± 7.6 days) in regimen 1 was shorter than 23.9 ± 12.6 days in regimen 2 (P < 0.01). In complete response cases at 7 weeks and the end of therapy, injection numbers and dosage in regimen 1 were more than those in regimen 2 at 7 weeks and the end of therapy (P < 0.05 and P < 0.01). However, to obtain complete response required the same dosage of steroid in two regimens at 7 weeks and the end of therapy according to calculation of steroid dose/lesion volume (mg/cm^3^) (Table 4).

**Table 4:** Influence of Dosage and Frequency in Clinical Effects of Steroid Treating Pretibial Myxedema

	Regimen 1 (N = 55)	Regimen 2 (N = 55)	Statistical analysis	
	Intralesional steroid once every 3 days	Intralesional steroid once every 7 days	Test	P value
Clinical effect occurring after injection (days), mean ± SD (min - max)	2.5 ± 0.6 (1 - 4)	3.1 ± 0.8 (1 - 7)	t = -4.629	0.000
Complete response time at 7 weeks (days), regimen 1 = 46, regimen 2 = 49	14.9 ± 7.6 (3 - 30)	23.9 ± 12.6 (7 - 49)	t = -4.230	0.000
Injection number at 7 weeks, regimen 1 = 46, regimen 2 = 49	4.2 ± 2.4 (1 - 8)	3.2 ± 1.7 (1 - 7)	t = 2.343	0.021
Dose of steroid at 7 weeks (mg), regimen 1 = 46, regimen 2 = 49	254 ± 197	140 ± 120	t = 3.424	0.001
Steroid dose/lesion volume at 7 weeks (mg/cm^3^), regimen 1 = 46, regimen 2 = 49	3.6 ± 4	3.7 ± 3.6	t = -0.231	0.818
Dose of steroid at end of therapy (mg), regimen 1 = 48, regimen 2 = 50	283 ± 240 (16 - 1,050)	163 ± 203 (11 - 1,300)	t = 2.674	0.009
Steroid dose/lesion volume at end of therapy (mg/cm^3^), regimen 1 = 48, regimen 2 = 50	3.5 ± 3.9	3.7 ± 3.6	t = -0.223	0.824

### Influence of PTM variants in clinical effects of steroid

**Figure 5:**
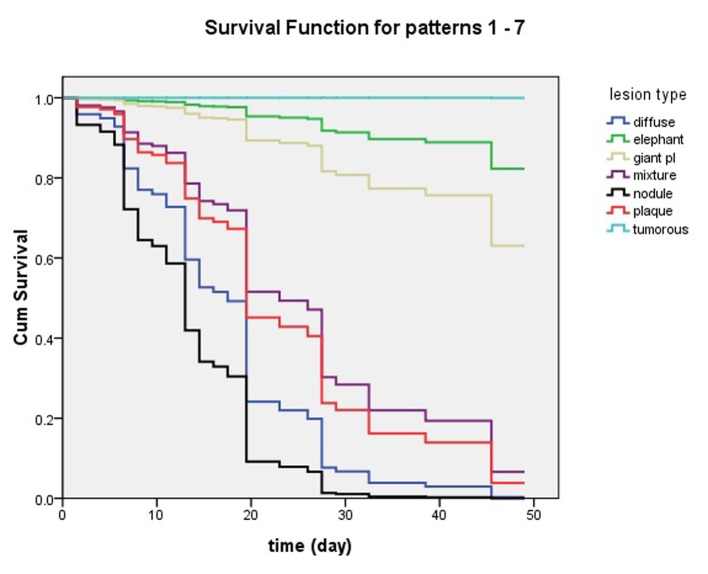
Influence of pretibial myxedema variants in clinical effects of intralesional steroid treating pretibial myxedema analyzed by Cox’s proportional hazards regression model. At 7 week, lesions of patients with nodule and diffuse swelling variants had disappeared and they had obtained 100% complete response. The patients with plaque and mixture variants had more than 90% complete response. The four variants of pretibial myxedema had excellent response to the therapy of intralesional steroid. However, less than 40% of the patients with giant plaque had complete response and more than 60% of them had no complete response. What’s worse, the elephantiasis variant had less than 20% of complete response and the tumorous variant had no complete response.

In the trial, we observed the complete response rate of non-severe variants was 95.8% (45/47) in regimen 1 and 100% (47/47) in regimen 2, but the severe was only 37.5% (3/8) in both regimens at 7 weeks and the end of therapy. There was no statistical difference between two regimens. However, the complete response rate of non-severe variants was significantly higher than that of severe variants (Fig. 5).

### Recurrence of PTM in the 3.5-year follow-up

In the follow-up, the recurrence rate of PTM went up with the extension of follow-up time, but there was no statistical difference between two regimens. At 3.5 years follow-up, the recurrence rate of PTM was 31.25% (15/48) and 32% (16/50) in regimens 1 and 2, respectively.

## Discussion

PTM is characterized by non-pitting thickening of local skin with autoimmunity, hyperplasia and infiltration. The proliferated fibrous connective tissues with unfettered hyaluronan synthesis and deposits not only infiltrate the subcutaneous adipose but make the dermis combine rich water to remodel the appearance of skin. The perivascular inflammatory infiltration with CD4^+^ and CD8^+^ T cells, coupled with increased levels of serum thyrotropin receptor autoantibody (TRAb), supports that cell-mediated and humoral immunity participate in the pathogenesis of PTM [1, 4, 5]. Glucocorticoids have the potent properties of anti-inflammation, inhibiting hyaluronan synthesis and making the skin atrophy [11, 12]. Theoretically, it is the glucocorticoids not the other immunosuppressants to be most suitable and effective medicine for treating PTM. Gimlette [13] reported oral large dosage of prednisone only produced transient efficacy but local injection of hydrocortisone produced permanent effect. Schwartz et al [8] reported the efficacy of topical glucocorticoid with compressive dressings exceeded topical glucocorticoid alone. The remission rate with local injection was 27.3%, greater than topical glucocorticoid or compressive dressings. Lang et al [14] reported the remission rate was 77.8% with intralesional injections of triamcinolone acetonide once a month. We found shorter interval time was needed between two injection sessions in order to obtain better effect [5]. Additionally, administration of triamcinolone acetonide is once a week in the instructions (package insert). However, its clinical effect with local injection was seen in 2 - 5 days in our experiences. So we design two frequency regimens: once every 3 days and once every 7 days. To our knowledge, this is the largest, prospective, randomly controlled trial of steroid treating PTM in the world. We have assessed the efficacy and safety of intralesional glucocorticoid injection treating PTM at 3 weeks, 7 weeks and the end of therapy.

In the trial, 110 patients were randomly distributed into regimens 1 and 2. The age, sex, occupation and clinical characteristics at baseline had no statistical difference between two regimens (P > 0.05). In order to keep the balance of lesional severity between two regimens, random distribution was stratified according to severe variants and non-severe variants of PTM. As a result, there were eight severe variant cases and 47 non-severe variant cases in each regimen. The severe variant cases consisted of one in tumorous, three in giant plaque and four in elephantiasis in each regimen. The 47 cases of non-severe variants were 24 in diffuse swelling, nine in mixture, eight in nodule and six in plaque in regimen 1, but in regimen 2, the non-severe variants were 25 in diffuse swelling, 13 in nodule, five in plaque and four in mixture. There was no statistical difference between two regimens. Also there were no statistical differences in the course and lesion numbers between two regimens. However, the size of PTM lesions was not easy to distribute evenly in two regimens but we could resolve this problem through comparing the decreased amounts of area, depth and volume of lesions at 3 weeks, 7 weeks and the end of therapy between regimens 1 and 2.

When we finished the trial, regimen 1 had obtained 87.3% complete response rate and regimen 2 had gotten 90.9%. Totally, the complete response rate was 89.1% (98/110). This complete response rate (89.1%) was higher than that (77.8%) in intralesional injection once a month [14], significantly higher than those in topical steroids [8]. However, the complete response rate in regimen 1 was higher than that in regimen 2 at 3 weeks after therapy, but no difference at 7 weeks after therapy. The clinical effect occurring time and complete response time in regimen 1 were significantly shorter than those in regimen 2 (P < 0.01), but injection numbers and steroid dose in regimen 1 were more than those in regimen 2. These further demonstrate the efficacy of glucocorticoid depends on the dose and frequency of injection *in vivo* [15]. Unfortunately, regimen 1 had earlier and more adverse reactions than regimen 2 in the trial. So we conclude that intralesional glucocorticoid could treat PTM and regimen 2 is generally better than regimen 1 in the treatment of PTM.

Through analyzing by Cox’s proportional hazards regression model, we found PTM variants influenced the efficacy of intralesional steroid injection for the therapy of PTM. Non-severe variants were easy to obtain complete response but severe variants were not. Because tumorous and giant plaque variants were resistant to the treatment of intralesional steroid, we recently reported that surgery combined with subcutaneous steroid injection could make patients get complete response [16]. However, there remained no effective method to treat the elephantiasis variant which was resistant to the intralesional steroid injection. On the other hand, severe variants progressed from non-severe variants [17], and severe variants could even make patients disabled. So it is necessary for the PTM patients to receive early treatment [18].

At 3.5-year follow-up, recurrent rates were 31.25% and 32% in regimens 1 and 2, respectively. There was no statistical difference in recurrent rates between two regimens. The data demonstrated that recurrent rate of PTM was not related to injection frequency of steroid. It could be related to the status of patients’ autoimmunity and local injury [19]. The role of local injury and serum TRAb levels in the pathogenesis of PTM recurrence should further be investigated to prevent PTM from recurrence.
